# Alfred M. Leonard

**Published:** 1857-03

**Authors:** 


*Died*, in Clarkson, Dec. 13th, 1856, ALFRED M. LEONARD, M. D., of congestion of the lungs.

Dr. Leonard was born in Woodstock, Vt., in 1802; he commenced the study of medicine in 1820, with his brother, Dr. John Leonard, in York, Livingston Co.; graduated at Bowdoin College, Me., in the spring of 1828, and commenced the practice of his profession in Clarkson, in September of the same year, where he continued until his death. By his medical science and skill in surgery he had won for himself a wide and enviable reputation.

---

*Buffalo Medical Association.*—The April session of this association, will be its first annual meeting under its corporation, at which time the election of officers will take place. At the March meeting, the subject of obtaining more commodious rooms for its occupancy, was brought up, and the matter was referred to the Trustees, with power to act according to their discretion in the matter. Notice was also given, of an amendment to the By-law to be acted upon at the next meeting, increasing the annual dues of the association from two dollars to three dollars per year, to meet the increased expenses which will accrue if larger rooms are taken. It is therefore desirable that there should be a full attendance of members on that occasion. In the meantime, we understand that the Treasurer is desirous of seeing all those who are in arrears for past dues and subscriptions, at his office, and have them liquidate all outstanding claims. The society certainly should be able to meet all demands against it at the expiration of the year.

